# Efficacy and safety of finerenone in IgA nephropathy: an observational multicentre study

**DOI:** 10.1093/ckj/sfaf125

**Published:** 2025-04-28

**Authors:** Qiao-Rui Wang, Longlong Wu, Jian Huang, Hong Pan, Xin-Fei Wang, Li Li, Fei Han, Yong-Fei Wang, Miao-Lian Wu, Yi Yang

**Affiliations:** Department of Nephrology, Center for Regeneration and Aging Medicine, the Fourth Affiliated Hospital of School of Medicine and International School of Medicine, International Institutes of Medicine, Zhejiang University, Yiwu, Zhejiang, China; Department of Nephrology, Center for Regeneration and Aging Medicine, the Fourth Affiliated Hospital of School of Medicine and International School of Medicine, International Institutes of Medicine, Zhejiang University, Yiwu, Zhejiang, China; Department of Nephrology, Jinhua Municipal Central Hospital, Jinhua, China; Department of Nephrology, Center for Regeneration and Aging Medicine, the Fourth Affiliated Hospital of School of Medicine and International School of Medicine, International Institutes of Medicine, Zhejiang University, Yiwu, Zhejiang, China; Department of Nephrology, Center for Regeneration and Aging Medicine, the Fourth Affiliated Hospital of School of Medicine and International School of Medicine, International Institutes of Medicine, Zhejiang University, Yiwu, Zhejiang, China; Department of Nephrology, Center for Regeneration and Aging Medicine, the Fourth Affiliated Hospital of School of Medicine and International School of Medicine, International Institutes of Medicine, Zhejiang University, Yiwu, Zhejiang, China; Kidney Disease Center, First Affiliated Hospital, Zhejiang University School of Medicine, Hangzhou, Zhejiang, China; School of Medicine and Warshel Institute for Computational Biology, Chinese University of Hong Kong, Shenzhen, Guangdong, China; Department of Paediatrics and Adolescent Medicine, University of Hong Kong, Hong Kong, China; Department of Pharmacy, The Fourth Affiliated Hospital, Zhejiang University School of Medicine, Yiwu, Zhejiang, China; Department of Nephrology, Center for Regeneration and Aging Medicine, the Fourth Affiliated Hospital of School of Medicine and International School of Medicine, International Institutes of Medicine, Zhejiang University, Yiwu, Zhejiang, China; Zhejiang-Denmark Joint Laboratory of Regeneration and Aging Medicine, Yiwu, Zhejiang, China

**Keywords:** efficiency, finerenone, IgA nephropathy, proteinuria, safety

## Abstract

**Background:**

Finerenone, a non-steroidal mineralocorticoid receptor antagonist, reduces renal risks in type 2 diabetic nephropathy, but its use in immunoglobulin nephropathy (IgAN) lacks evidence. This study assessed the safety and efficacy of 6-month finerenone treatment in IgAN patients.

**Methods:**

This retrospective cohort study was mainly conducted in three Grade 3A hospitals. Patients diagnosed with IgAN and receiving standard supportive care were included. Participants were divided into the renin–angiotensin system inhibitor (RASI) and RASI + finerenone groups. The primary outcome was the percentage decrease in protein-to-creatinine ratio (PCR) over 6 months following the index study visit.

**Results:**

In total, 178 patients were included in the analysis. PCR was reduced by 45.1% in the RASI + finerenone group and 32.5% in the RASI group (*P* = .013). Compared with 18 patients (20.2%) in the control group, 33 (37.1%) had residual PCR reduced to <0.3 g/g. After 6 months, serum potassium increased by 0.17 mmol/L from baseline, with no uncontrollable hyperkalemia (persistent serum potassium >5.5 mmol/L despite treatment). In addition, one patient presented with a blood pressure <90/60 mmHg without significant clinical symptoms in the RASI + finerenone group. And eGFR decreased by 1.94 ± 6.73 mL/min/1.73 m^2^ from baseline, but not statistically significant. There were no differences in the incidence of adverse events between the two groups.

**Conclusions:**

Finerenone added to optimized RAS blocker therapy significantly reduced PCR in IgAN patients, and its safety profile was consistent with previous reports, suggesting the need for long-term renal outcome studies.

**Trial registration:**

ClinicalTrials.gov NCT06460987

## Graphical Abstract

**Graphical Abstract:**
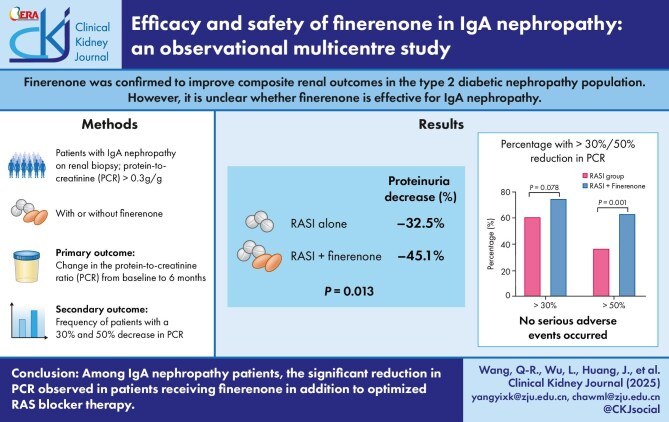

KEY LEARNING POINTS **What was known:**

- In the FIDELIO-DKD and FIGARO-DKD results, finerenone was confirmed to improve composite renal outcomes in the type 2 diabetic nephropathy population; finerenone has anti-inflammatory and anti-fibrotic effects by blocking mineralocorticoid receptors.
- Immunoglobulin A nephropathy (IgAN) is the most common glomerular disease and one of the most common reasons for young people to require renal replacement.
- Based on its mechanism, finerenone may also be useful in the treatment of IgAN.

**This study adds:**

- The primary endpoint was the percentage change in PCR from baseline to 6 months.
- Finerenone can significantly reduce proteinuria in patients with IgAN in the short term, and it appears relatively safe.

**Potential impact:**

- This study suggests that finerenone has the effect of reducing urinary protein in IgAN, providing support for conducting larger clinical studies.

## INTRODUCTION

Immunoglobulin A nephropathy (IgAN) is the most common glomerular disease globally [1] and a leading cause of kidney transplantation in young adults. Studies show that up to 11.2% of patients progress to end-stage renal disease (ESRD) within 5 years [2].

The KDIGO (Kidney Disease: Improving Global Outcomes) 2021 Guidelines recommend controlling blood pressure and reducing proteinuria to slow kidney disease progression. For proteinuria >0.5 g/day, angiotensin-converting enzyme inhibitors (ACEIs) or angiotensin II receptor blockers (ARBs) are first-line treatments [3]. Proteinuria <0.3 g/day is often considered complete remission [4, 5]. Glucocorticoids may be used for high-risk patients, but risks and benefits must be weighed [6]. Optimized supportive care remains the mainstay of treatment.

A large clinical study confirmed that finerenone reduces the risk of composite renal outcomes in patients with type 2 diabetic nephropathy [7, 8]. Results from the MinerAlocorticoid Receptor Antagonist Tolerability Study (ARTS) showed that finerenone causes less hyperkalaemia than spironolactone [9]. As a non-steroidal mineralocorticoid receptor antagonist (MRA), finerenone has anti-inflammatory and anti-fibrotic effects with minimal impact on serum potassium. While its anti-fibrotic properties may protect kidney function in various conditions, it has been primarily studied in diabetic nephropathy.

It is unknown whether finerenone works for IgAN. Based on safety data from the FIDELITY analysis, which showed outcomes similar to placebo, we aimed to assess the short-term efficacy and safety of finerenone in IgAN patients.

## MATERIALS AND METHODS

### Study design and patients

This was a retrospective, noninterventional, multicentre, observational cohort study. All patients attending the nephrology clinic between December 2022 and February 2024 were initially screened from electronic health records, and manually collected patients with IgAN confirmed by renal biopsy. Clinical records and laboratory results were collected between March 2024 and May 2024. For all participants in the renin–angiotensin system inhibitor (RASI) group, baseline (‘Time 0’) was operationally defined as the first visit within the study window during which they fulfilled all inclusion criteria, and that of the patients in RASI + finerenone group as the time when they started using finerenone. We continued to collect data for the next 1, 2, 3 and 6 months. The inclusion criteria were as follows: age between 18 and 75 years, receiving at least 3 months of maximum dose RASI therapy according to the KDIGO glomerulonephritis guideline [3] serum potassium level <5 mmol/L, and protein-to-creatinine ratio (PCR) >0.3 g/g. The exclusion criteria included patients currently receiving systemic immunosuppressive therapy, those with secondary IgAN, polycystic kidney disease, lupus nephritis, previous renal transplantation, malignant tumours, heart failure with an ejection fraction <40%, and those undergoing renal replacement therapy or with an estimated glomerular filtration rate (eGFR) <25 mL/min.

This study was conducted at the Fourth Affiliated Hospital of Zhejiang University School of Medicine, the First Affiliated Hospital of Zhejiang University School of Medicine and Jinhua Municipal Central Hospital Medical Group. This study was approved by the Ethics Committee of the Fourth Affiliated Hospital of Zhejiang University School of Medicine. The trial was registered at ClinicalTrials.gov (number NCT06460987).

### Procedures

This study had a 6-month observation period. Finerenone, though off-label, was used in IgAN patients with suboptimal proteinuria control at three hospitals, based on its benefits in diabetic nephropathy and patient consent. It was recommended if proteinuria remained >0.3 g/day despite maximum ACEI/ARB doses. Patients received 10 mg/day of finerenone, with creatinine and potassium levels checked after 2 weeks to adjust the dose and ensure safety.

The dose is increased to 20 mg/day if tolerated by the patient. The specific study period was the first visit when patients met the study inclusion criteria and the following 1, 2, 3 and 6 months. GFR was estimated using the Chronic Kidney Disease Epidemiology Collaboration Creatinine Equation (www.kidney.org/professionals/gfr_calculator), and the results presented using the CKD-EPI creatinine equation. The assessments included body mass index, age, pathological data (MEST-C scores based on the Oxford classification of IgAN), PCR, serum creatinine, eGFR, uric acid, haemoglobin, haematocrit and blood pressure. Hyperkalemia was defined as serum potassium >5.5 mmol/L. Uncontrollable hyperkalemia was defined as serum potassium levels persistently >5.5 mmol/L despite adherence to standard management strategies, including dietary potassium restriction, diuretic use or dose reduction of relevant medications. Uncontrollable adverse reactions were defined as severe or intolerable side effects that led to the discontinuation of the treatment, regardless of other interventions.

In retrospective studies, blood pressure classification is primarily based on clinical consultation records from outpatient visits, which lacked specific blood pressure measurement values. Thus, the therapeutic effects of medications can only be assessed through qualitative descriptions. Blood pressure levels were categorized based on the definitions of hypotension and KDIGO-recommended control targets [3]. Hypotension was defined as blood pressure <90/60 mmHg (Grade A). Conversely, a blood pressure >130/80 mmHg (Grade C) indicated inadequate blood pressure management.

### Outcomes

The primary endpoint was the percentage decrease in PCR from baseline to 6 months. Secondary outcomes included the proportion of patients achieving a 30% or 50% reduction in PCR and PCR <0.3 g/g at 6 months. We also assessed the impact of previous immunosuppressive therapy and concomitant use of sodium-glucose cotransporter 2 inhibitors (SGLT2i). Safety analysis evaluated serious adverse events, eGFR declines >30% or 40%, and hyperkalemia.

### Statistical analysis

Normally distributed data are shown as mean ± standard deviation; non-normally distributed data as median (interquartile range); and categorical data as counts and percentages. Missing values were handled using multiple imputation, creating 20 datasets with plausible values based on prediction equations. Statistical analyses were performed on each dataset, and results were combined for a summary estimate [10–12]. Sensitivity analyses using worst-case imputation, last observation carried forward (LOCF), and mean imputation were conducted to test robustness (see Supplementary materials). To minimize intergroup differences, propensity score matching (PSM; 1:1 ratio) was performed with a calliper value of 0.03, based on covariates like age, sex, baseline PCR, eGFR, hypertension history, MEST-C score, SGLT2i use, biopsy time and diabetes. The main analysis was performed on both the per protocol set (PPS) and the full analysis set (FAS) (intention-to-treat-principle). We used the difference-in-difference method (DID) and generalized estimating equations (GEE) to evaluate the effect of adding finerenone on the percentage change in PCR. DID eliminates the confounding effects of time trends and between-group differences by comparing the differences between the intervention and control groups before and after the intervention. GEE provides robust parameter estimates by adjusting for correlations in longitudinal data. GEE incorporates covariates similar to those in PSM, along with the factors of time and the interaction between treatment and time. Comparisons were made by fitting an ANOVA model to the data at 6 months, including the treatment group, baseline proteinuria and follow-up time as covariates in a nested PCR, with results presented as point estimates (least-squares mean) and confidence intervals (CIs). Simple ratio calculations were utilized to estimate the number of patients who achieved a 30% or 50% reduction in proteinuria at each time point.

To assess the effects of finerenone and SGLT2i on the percentage decrease in (PCR), we constructed multivariate linear regression models adjusted for covariates including age, sex, baseline eGFR, diabetes status and baseline PCR. The primary outcome analysis was conducted again according to the different levels of PCR and eGFR. As a *post hoc* analysis, we compared whether patients with a prior use of immunosuppressants (e.g. glucocorticoids, mycophenolate mofetil, tacrolimus and *Tripterygium wilfordii*) could benefit more from the addition of finerenone to intensive supportive care. Pearson's correlation coefficients between the percentage decrease in PCR at 6 months and the change in eGFR from baseline to 6 months were calculated for all groups. Changes in clinical indicators from baseline to 6 months were also compared. Statistical significance was set at *P* < .05. All analyses were performed using SPSS version 25 and R version 4.4.2.

## RESULTS

### Baseline characteristics

**Figure 1:**
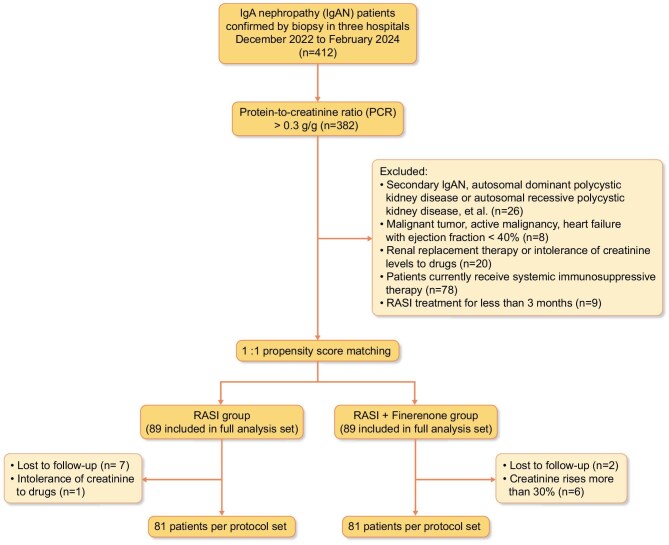
Flow chart of the study.

A total of 382 patients were included in this study (Fig. 1). Patients were divided into two groups: the RASI group and the RASI + finerenone group (all details are shown in Supplementary data, Table S1).

After PSM, the standardized differences in all covariates used were <0.1 (Supplementary data, Fig. S1). There were 178 patients in FAS, and 18 patients did not meet the criteria for per-protocol analysis for reasons outlined in the study flow chart (Fig. 1). These data were statistically comparable, achieving baseline equilibrium. In FAS population, 33.7% and 30.3% of patients had PCR levels > 1 g/g. The average PCR in each group is as follows: 0.80 (0.58, 1.13) and 0.77 (0.61, 1.22). 44.9% (RASI group), and 46.9% (RASI + finerenone group) of patients were taking SGLT2i at the time of enrolment. And the dosage of medication remains stable (Table 1).

**Table 1:** Baseline characteristics of IgAN patients at the enrollment after matching.

	PPS population			FAS population		
	RASI group (*n* = 81)	RASI + F group (*n* = 81)	*P*	RASI group (*n* = 89)	RASI + F group (*n* = 89)	*P*
Age, years	39.00 (33.00, 45.00)	36.00 (32.00, 46.00)	.741	39.00 (33.00, 45.00)	36.00 (32.00, 48.00)	.949
Sex, male, *n* (%)	38 (46.9)	43 (53.1)	.432	40 (44.9)	50 (56.2)	.134
BMI, kg/m^2^	23.90 (20.97, 25.85)	25.13 (22.00, 26.81)	.143	23.90 (21.43, 26.00)	24.11 (22.30, 26.23)	.150
Hypertension, yes, *n* (%)	37 (45.7)	35 (43.2)	.752	40 (44.9)	39 (43.8)	.880
Hypertension hierarchy, 90/60 mmHg < BP < 130/80, *n* (%)	77 (95.1)	78 (96.3)	.902	84 (94.4)	83 (93.3)	.943
DM, yes, *n* (%)	7 (8.6)	6 (7.4)	.772	7 (7.9)	9.0 (10.1)	.787
Scr, μmol/L	98.00 (78.00, 121.00)	103.00 (75.82, 128.00)	.854	100.00 (78.00, 120.00)	99.00 (76.00, 124.00)	.833
eGFR, mL/min/1.73 m^2^	73.15 ± 24.51	73.21 ± 24.96	.987	74.47 ± 24.74	75.15 ± 25.21	.661
PCR, g/g	0.80 (0.58, 1.17)	0.76 (0.61, 1.09)	.225	0.80 (0.58, 1.13)	0.77 (0.61, 1.22)	.493
Albumin, g/L	41.15 ± 3.7	42.33 ± 3.17	.080	41.50 (38.70,43.99)	42.45 (40.15,43.90)	.086
Potassium, mmol/L	4.11 ± 0.36	4.15 ± 0.35	.469	4.13 ± 0.33	4.14 ± 0.38	.782
Blood glucose, mmol/L	4.78 ± 0.57	5.10 ± 0.83	.052	4.69 ± 0.44	4.98 ± 0.90	.067
Sodium, mmol/L	140.39 ± 2.19	140.91 ± 2.24	.133	140.23 ± 2.22	141.22 ± 2.23	.004
Haemoglobin, g/L	138.57 ± 19.77	138.51 ± 17.50	.982	140.00 (128.00 152.00)	139.00 (124.18 151.25)	.956
Haematocrit, %	41.50 ± 5.56	42.00 ± 4.97	.547	41.66 ± 5.39	41.79 ± 5.14	.876
Uric acid, μmol/L	360.93 ± 94.41	352.73 ± 83.48	.559	363.34 ± 94.49	349.49 ± 85.87	.322
Biopsy time, years	3.20 (2.00, 7.00)	3.00 (2.00, 5.00)	.215	3.50 (2.20, 7.00)	3.00 (2.00, 5.50)	.120
Oxford classification, *n* (%)						
M, M1	41 (50.6)	38 (46.9)	.637	45 (50.6)	40 (44.9)	.453
E, E1	6 (7.4)	8 (9.9)	.576	11 (12.4)	14 (15.7)	.518
S, S1	57 (70.4)	60 (74.1)	.599	59 (66.3)	63 (70.8)	.519
T			.774			.784
T1	18 (22.2)	20 (24.7)		20 (22.5)	24 (27.0)	
T2	1 (1.2)	2 (2.5)		2 (2.3)	2 (2.3)	
C			.272			.241
C1	20 (24.7)	27 (33.3)		23 (25.7)	31 (34.8)	
C2	0 (0.0)	1 (1.2)		0 (0.0)	1 (1.1)	
Hyperuricemia	17	16		19	20	
Patients with hepatitis B	10	6		10	6	
Hyperlipidemia	12	2		12	4	
Therapy with diuretics	0	0		0	0	
Therapy with SGLT2i, *n* (%)	37 (45.7)	38 (46.9)	.875	40 (44.9)	38 (46.9)	.797
Duration of medication, months	11.30 (3.50, 21.80)	10.30 (2.60, 20.00)	.576	10.00 (2.80, 19.80)	9.40 (3.40, 22.40)	.772
Therapy of ACEI/ARB						
≥50% of maximum labelled dose, *n* (%)	50 (61.7)	45 (55.6)	.425	53 (60.2)	48 (53.9)	.425
Duration of medication, months	13.00 (3.00, 22.00)	11.00 (3.00, 23.00)	.776	12.00 (3.00, 21.00)	11.00 (3.00, 23.00)	.834
Previous immunosuppressive therapy, *n* (%)	33 (40.7)	37 (45.7)	.634	36 (40.5)	40 (44.9)	.544
Time to discontinuation immunosuppressant, months	12.72 (22.78)	18.11 (18.92)	.393	16.81 (20.14)	19.15 (15.27)	.412

Age, BMI, Scr, PCR, biopsy time are presented as median with interquartile range. Albumin, eGFR, potassium, sodium, blood glucose, haemoglobin, haematocrit, uric acid are presented as mean ± standard deviation. Categorical data as count and percentage.

RASI + F, RASI + finerenone; BMI, body mass index; BP, blood pressure; DM, diabetes mellitus; Scr, serum creatine; M, mesangial hypercellularity; E, endocapillary hypercellularity; S, segmental glomerulosclerosis; T, segmental glomerulosclerosis; C, cellular/fibrocellular crescents.

### Primary outcomes

**Figure 2:**
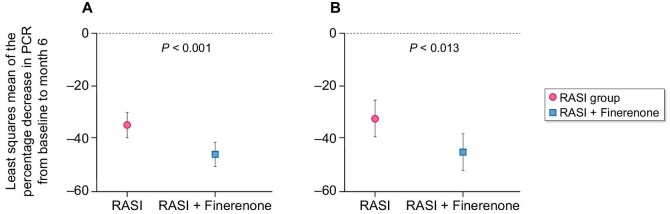
Percentage change in least squares mean PCR at Month 6 relative to baseline in patients treated with finerenone.

**Figure 3:**
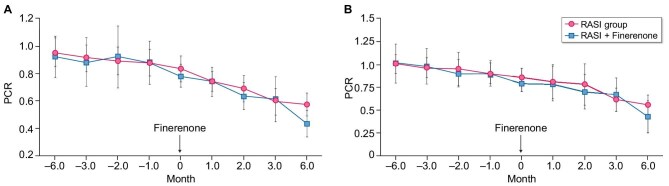
Time trends of PCR slopes in the RASI and RASI + finerenone groups before and after finerenone initiation.

The primary efficacy analysis was performed on the FAS. Ninety-seven percent of patients in the RASI + finerenone group received finerenone 10 mg once daily, with the remaining 3% taking a lower dose of 5 mg or a higher dose of 20 mg. All patients were maintained on an optimized and stable RAS blockade. In the primary outcome analysis, the generalized estimating equations adjusted for time and baseline PCR levels showed a significant effect of finerenone on the primary endpoint (*P* = .001). The least-squares mean of the percentage change in PCR from baseline to 6 months was lower in the RASI group than in the RASI + finerenone group [–32.5% (95% CI –39.5, –25.4) and –45.1% (95% CI –52.2, –38.1); *P* = .013] (Fig. 2). In the PPS, which excluded participants with major protocol deviations, the findings were consistent with the FAS, showing a similar trend with a slightly higher effect size (*P* < .001) (Table 2). DID analysis showed that after adding finerenone, the PCR slope of the intervention group decreased significantly [β = –0.172 (95% CI –0.231, –0.113); *P* < .001] (time trend diagram shown in Fig. 3). Sensitivity analysis confirmed the robustness of the primary results. We used different imputation methods to calculate the percentage of PCR decrease in the two groups, and the *P*-values were all <.05, which was consistent to the main analysis (Supplementary data, Table S4).

**Table 2:** The PCR at baseline and 6 months and the number of people with different levels of PCR.

	Patients in per protocol set			Patients in full analysis set		
	RASI group (*n* = 81)	RASI + F (*n* = 81)	*P*	RASI group (*n* = 89)	RASI + F (*n* = 89)	*P*
Percentage change in PCR, %	–34.7 (–39.5, –30.0)	–46.1 (–50.8, –41.4)	<.001	–32.5 (–39.5, –25.4)	–45.1 (–52.2, –38.1)	.013
At baseline						
Number of people with PCR >1 g/g, *n* (%)	28 (34.6)	24 (29.6)	.405	30 (33.7)	27 (30.3)	.634
PCR, g/g	0.80 (0.57, 1.17)	0.76 (0.61, 1.09)	.754	0.80 (0.58, 1.13)	0.77 (0.61, 1.22)	.493
At 6 months						
Number of people with PCR >1 g/g, *n* (%)	9 (11.1)	10 (12.3)	1	10 (11.2)	12 (13.5)	.82
Number of people with PCR <0.3 g/g, *n* (%)	17 (21.0)	32 (39.5)	.016	18 (20.2)	33 (37.1)	.002
PCR, g/g	0.50 (0.3, 0.75)	0.40 (0.24, 0.57)	.015	0.50 (0.30, 0.72)	0.40 (0.25, 0.57)	.020
Frequency of patients with 30% decrease in PCR	48 (59.3%)	61 (75.3)	.044	54 (60.7)	66 (74.2)	.078
Frequency of patients with 50% decrease in PCR	28 (34.6)	52 (64.2)	<.001	32 (36.0)	56 (62.9)	.001

The percentage changes in PCR are presented as least square means and corresponding confidence intervals.

Treatment effect is expressed as the percentage change in PCR compared with baseline.

RASI + F, RASI + finerenone.

Additionally, we compared the medication efficacy of patients previously on immunosuppressants. The percentage decrease in PCR was not statistically significant [–34.0% (95% CI –40.6, –27.4) and –42.1% (95% CI –48.3, –35.8); *P* = .083] (baseline characteristics, Supplementary data, Table S2; outcomes, Table 3). Linear regression modelling results showed that the percentage decrease in PCR was also significantly higher in patients using SGLT2i than in those not using SGLT2i (B = –0.17, standard error = 0.060, *P* < .001). However, we observed no significant interaction between the use of finerenone and SGLT2i use status (interaction term β = –20.3, standard error = 0.120, *P* = .091) (baseline characteristics, Supplementary data, Table S3; outcomes, Table 4). Specifically, in patients receiving finerenone, RASi and SGLT2i combination therapy, the PCR reduction from baseline to 6 months was 44.9% (95% CI –55.3, –34.4) compared with patients receiving RASi and SGLT2i alone [–36.7% (95% CI –47.4, –28.3)], but the difference was not found to be statistically significant (*P* = .050, show in Supplementary data, Fig. S2).

**Table 3:** Effect of finerenone on IgAN patients who have previously used immunosuppressants in the full analysis set.

	RASI group (*n* = 36)	RASI + F group (*n* = 40)	*P*
Percentage change in PCR, %	–34.0 (–40.6, –27.4)	–42.1% (–48.3, –35.8)	.083
At baseline			
Number of people with PCR >1 g/g, *n* (%)	15 (41.7)	12 (30.0)	.342
PCR, g/g	0.89 (0.57, 1.18)	0.74 (0.62, 1.29)	.743
At 6 months			
Number of people with PCR >1 g/g, *n* (%)	5 (13.9)	7 (17.5)	.759
Number of people with PCR <0.3 g/g, *n* (%)	10 (27.8)	15 (37.5)	.465
PCR, g/g	0.52 (0.28, 0.78)	0.45 (0.26, 0.59)	.435

The percentage changes in PCR were presented as least square means and corresponding CIs.

RASI + F, RASI + finerenone.

**Table 4:** Linear regression modelling results for percentage PCR decline.

	Regression coefficient (B)	Standard error	*P*
Treatment group (RASI + F vs RASI)	–0.289	0.060	.025
SGLT2i usage status (yes vs no)	–0.170	0.061	<.001
Interaction term (treatment group × SGLT2i use status)	–0.203	0.120	.091
Covariate			
Age (for each additional year)	–0.005	0.003	.087
Gender (male vs female)	0.018	0.067	.783
Baseline eGFR (per 1 mL/min/1.73 m² increase)	0.01	0.049	.062
Diabetes (yes vs no)	0.026	0.145	.858
Baseline PCR (per 1 g/g increase)	–0.064	0.040	.011
Hypertension (yes vs. no)	0.050	0.067	.463

RASI + F, RASI + finerenone.

In prespecified subgroup analyses, finerenone demonstrated a strong ability to reduce urinary protein, regardless of baseline eGFR level (<60 vs ≥60 mL/min/1.73 m^2^). Additionally, in the subgroup with low baseline proteinuria (protein excretion <1 g/g), the addition of finerenone to the maximally optimized therapy was statistically significant (Supplementary data, Table S5).

### Secondary outcomes

**Figure 4:**
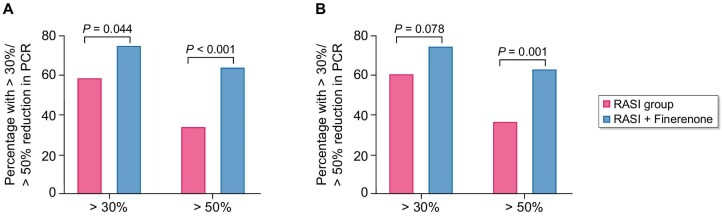
Percent with >30% or >50% reduction in PCR during follow-up in two groups.

In the secondary outcome analysis, at 6 months, 66 (74.2%) patients in the RASI + finerenone group showed a 30% decrease compared with 54 of 89 (60.7%) patients in the RASI group (*P* = .078). The RASI + finerenone group demonstrated a significant advantage over the RASI group in achieving a 50% reduction in PCR (*P* = .020; Fig. 4 and Table 2). Residual PCR persisted ≥1 g/g or decreased to <0.3 g/g in 12 (13.5%) and 33 (37.1%) patients, respectively, compared with 10 (11.2%) and 18 (20.2%) patients, respectively, in the RASI group (Table 2).

Supplementary data, Table S6 lists the changes in clinical indicators. Serum creatinine decreased by 8.6 µmol/L (*P* = .040) in the RASI group and increased by 5 µmol/L in the RASI + finerenone group (*P* = 0.31). Regarding serum potassium, there was a significant increase in the RASI + finerenone group (serum potassium at 6 months was 4.31 mmol/L, with a difference of 0.17 mmol/L from baseline, *P* = .03), but causing no severe adverse events. *Post hoc* analyses showed that no meaningful correlation was observed between the percentage decrease in PCR and changes in eGFR (PCCs (Pearson correlation coefficient) = –0.057, *P* = .613 and PCCs = 0.105, *P* = .351) (Supplementary data, Fig. S3).

### Safety and adverse events

There was no difference in the overall incidence of adverse events and serious adverse events between the RASI group and the RASI + finerenone group (Table 5). Adverse events occurred in 21 patients in the RASI + finerenone group. Urinary tract infections, seen in patients on SGLT2i, improved with clinical interventions. Hypotension occurred in four RASI patients and one RASI + finerenone patient, but all improved after adjusting RASI doses. In the RASI + finerenone group, six patients discontinued treatment due to a >30% increase in creatinine, with one also having potassium >5.5 mmol/L. In the RASI group, one patient stopped treatment due to elevated creatinine levels (*P* = .118). One patient developed hyperkalaemia at 20 mg finerenone, which resolved after dose reduction. Six patients had upper respiratory infections that improved without antibiotics. Other adverse reactions were managed by reducing medication doses.

**Table 5: Serious adverse events:** and adverse events in RASI group and RASI + F group.

Adverse	RASI group (*n* = 89)	RASI + F group (*n* = 89)
Serious adverse events, *n*	0	0
No. of adverse events, *n* (%)		
1	17 (19.1)	20 (22.5)
≥2^a^	0	2 (2.2)
Creatinine exceeds safety limit^c^, *n* (%)	1 (1.1)	6 (6.7)
Urinary tract infection^a^,^b^, *n* (%)	7 (7.9)	4 (4.5)
Hypotension^b^, *n* (%)	4 (4.5)	4 (4.5)
Hyperkalemia^c^, *n* (%)	0	2 (2.2)
Upper respiratory tract infection, *n* (%)	5 (5.6)	6 (6.7)

Creatinine exceeds safety limit: in the RASI + F group, it refers to a 30% increase in serum creatinine over baseline in patients receiving finerenone, and in the RASI group, it means that the patient's creatinine level is no longer suitable for continuing to use the drug.

a All patients with urinary tract infection were taking SGLT2i orally.

^b,c^One patient experienced both adverse events.

RASI + F, RASI + finerenone.

## DISCUSSION

This study shows that finerenone reduces proteinuria in IgAN patients. While most trials focus on high-risk CKD patients with proteinuria >1 g/day [5, 13], intensive immunosuppressive therapy often causes significant side effects [6]. According to the KDIGO guidelines, supportive care remains the primary treatment for IgAN [3]. Finerenone, a non-steroidal MRA [14, 15], has stronger receptor affinity and lower hyperkalaemia risk compared with spironolactone and eplerenone, while maintaining similar sodium-lowering effects [16]. The FIDELIO-DKD (The Finerenone in Reducing Kidney Failure and Disease Progression in Diabetic Kidney Disease) trial showed finerenone reduces progression risk in type 2 diabetes by 18% [7]. In our study, finerenone reduced PCR by 45.1%, with more patients achieving >50% proteinuria reduction. This may be due to reduced glomerular pressure from sodium excretion. MR antagonism also reduces kidney oxidative stress and fibrosis [17].

High proteinuria is strongly linked to kidney and cardiovascular risks, regardless of eGFR [18]. While significant proteinuria is often defined as >1 g/day, levels >0.3 g/day can also predict poor kidney outcomes [5]. Early proteinuria reduction lowers long-term kidney risks [19]. Evidence for ACEIs/ARBs in IgAN comes from small trials with inconsistent benefits, and no studies show they slow kidney failure progression [20, 21]. Immunosuppressants also fail to prevent eGFR decline or ESRD long-term [6, 22].

In this study, 40.5% and 44.9% of patients had prior immunosuppressive therapy (discontinued 17 and 19 months earlier, respectively). A 6-month steroid treatment can reportedly reduce proteinuria levels in the long term [23]. We found no statistical difference between patients with or without prior immunosuppressant use. Subgroup analysis showed better efficacy in patients with lower baseline proteinuria, likely because corticosteroids or immunosuppressants are not superior to supportive care in IgAN patients with mild proteinuria (<1 g/g) [24].

The renoprotective effects of ACEIs can be divided into haemodynamic and non-haemodynamic effects [25]. Early studies show ACEIs significantly lower blood pressure, and even adjusting diastolic pressure can benefit kidney function in patients with high proteinuria. [26]. However, it is difficult to separate blood pressure-lowering effects from ACEIs’ unique kidney benefits. Long-term ACEI use provides durable renoprotection. In addition, Wolf [27] *et al*. also suggested that high-risk patients with proteinuria >1 g/day continue to benefit from long-term ACEI therapy. This may explain why, in our study, despite stable RASI use before enrolment, PCR still decreased by 32.5% after 6 months.

The FIGARO-DKD (Finerenone in Reducing Cardiovascular Mortality and Morbidity in Diabetic Kidney Disease) and FIDELIO-DKD studies reported that most hyperkalemia events were mild to moderate. In our study, serum potassium increased by 0.17 mmol/L after 6 months, a known side effect of MR antagonism [16]. The 2022 KDIGO guidelines recommend managing hyperkalemia with potassium-lowering measures before reducing or stopping medication [28]. Patients with poorer kidney function are more prone to hyperkalemia due to impaired potassium metabolism [29]. In our study, two patients developed hyperkalemia: one discontinued due to elevated creatinine, while the other normalized potassium after dose adjustment.

Our study found that both finerenone and dapagliflozin alone significantly reduced proteinuria, but multiple regression analyses showed no significant benefit from their combination. This contrasts with Mårup *et al*., who reported a significant reduction in albuminuria with the combination [30]. SGLT2 inhibitors lower proteinuria by reducing intraglomerular pressure and improving metabolic disorders, while finerenone works through anti-inflammatory and antifibrotic effects [31]. The inconsistent results may stem from varying synergistic or antagonistic effects in different patient populations. Both studies involved patients with baseline proteinuria <1 g/g, and the smaller sample size may have influenced the response to combination therapy.

No eGFR changes were observed in the RASI + finerenone group, likely due to the short 6-month follow-up. While MRAs may cause an initial eGFR decline, long-term eGFR typically stabilizes [32]. The KDIGO guidelines emphasize the cardio-renal benefits of such treatments and advise against discontinuing therapy due to early, reversible eGFR declines [28].

This study offers a new treatment approach for IgAN. No serious adverse events, like uncontrolled hyperkalemia or hypotension, were reported in finerenone-treated patients. Systolic blood pressure changes stayed within 30 mmHg, and no patients stopped medication due to low blood pressure.

Several limitations of this study should be acknowledged. First, the observational design risks residual confounding despite multivariate adjustment for baseline proteinuria, eGFR, and RASI use. Second, the 6-month median follow-up precludes assessment of composite endpoints (40% eGFR decline/kidney failure) requiring extended observation. Third, the limited sample size (*n* = 178) constrains subgroup analysis power. These methodological constraints are counterbalanced by the ongoing phase III FIND-CKD trial [33], which in a 32-month randomized controlled trial of >400 biopsy-proven IgAN patients, with the primary endpoint being maintenance of the eGFR slope. We look forward to the findings of this trial.

## CONCLUSION

In conclusion, among patients with IgAN, the significant reduction in PCR observed in those receiving finerenone alongside optimized RASI inhibitor therapy, without evidence of uncontrollable adverse events, suggests the need for long-term studies investigating clinical endpoints.

## Supplementary Material

sfaf125_Supplemental_Files

## Data Availability

Upon submission, authors agree to make any materials, data and associated protocols available upon request.
