# Supplementary material for: Efficacy and safety of finerenone in IgA nephropathy: an observational multicentre study
Source: Clin Kidney J. 2025 Apr 28;18(5):sfaf125. doi: 10.1093/ckj/sfaf125 (PMC12067071; doi:10.1093/ckj/sfaf125)

Figure S1: Visualization of the standardized mean difference after propensity score matching.

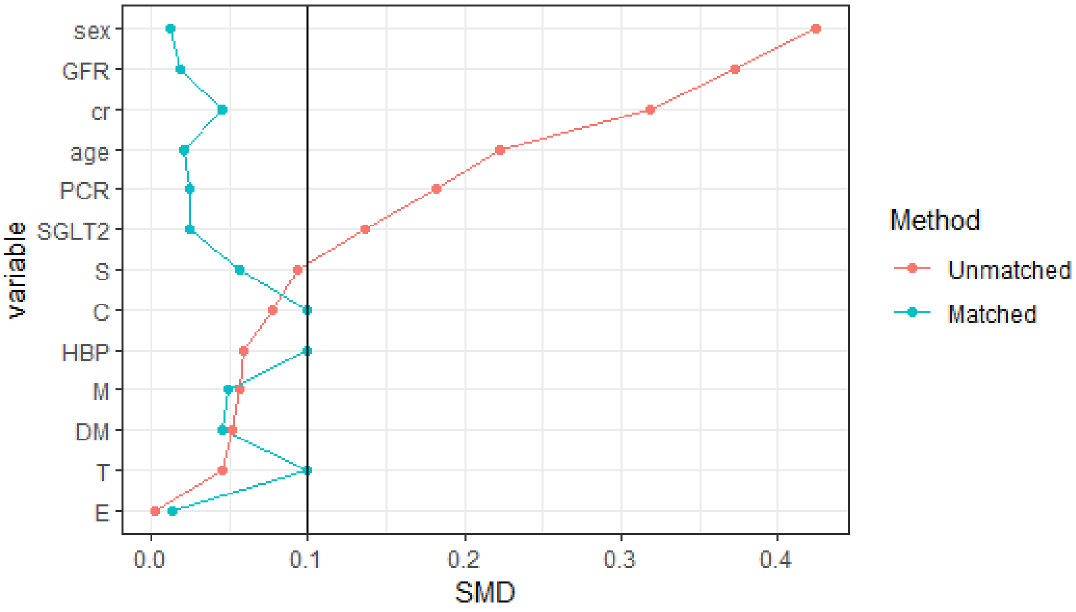

Abbreviations: GFR, glomerular filtration rate; PCR, protein-to-creatinine ratio; SGLT2, Sodium-Glucose Cotransporter 2 inhibitor; M, Mesangial hypercellularity (M0/M1, <or equal to >50% of glomeruli with >4 mesangial cells/area); E, Endocapillary hypercellularity (E0/E1, absent/present); S, Segmental glomerulosclerosis (S0/S1, absent/present); T, Tubular atrophy/interstitial fibrosis (T0/T1/T2, <25%, 25−50%, >50%). C, Cellular/fibrocellular crescents (C0/C1/C2, absent or crescents in a least 1 but <25% of glomeruli or crescents in at least 25% of glomeruli); HBP, high blood pressure; DM, diabetes.

After matching, the SMD values of all covariates (age, sex, baseline PCR, baseline eGFR, history of hypertension, MEST-C score, use of SGLT2i, and diabetes) were less than 0.1.

Figure S2: Percentage change in least squares mean PCR at month 6 relative to baseline in patients treated with finerenone and SGLT2i

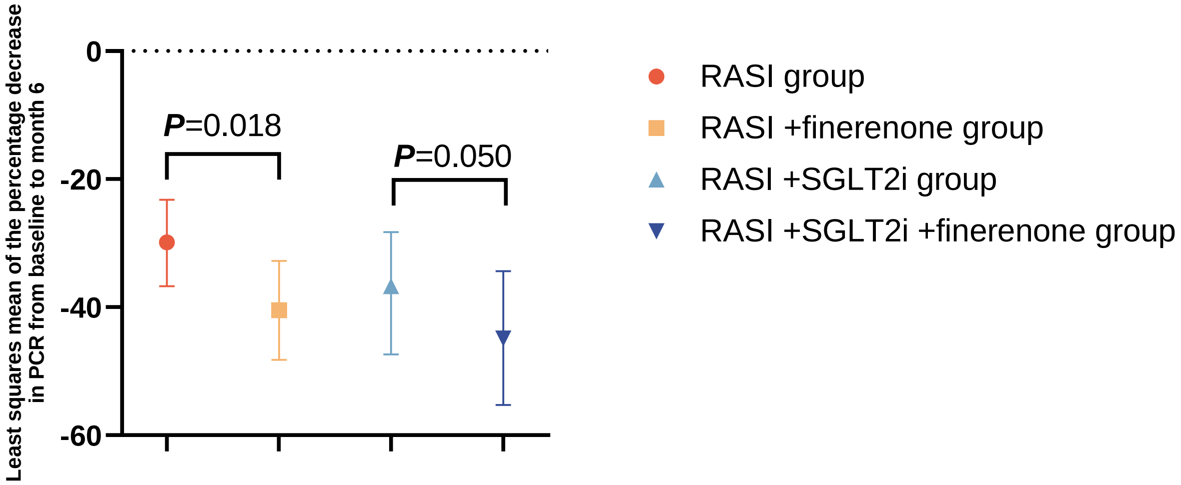

Abbreviations: PCR, protein-to-creatinine ratio; SGLT2 inhibitors, Sodium-Glucose Cotransporter 2 Inhibitor.

Figure S3: Scatterplot for percentage change to baseline in protein-to-creatinine ratio (PCR) and Change from Baseline in estimated glomerular filtration rate

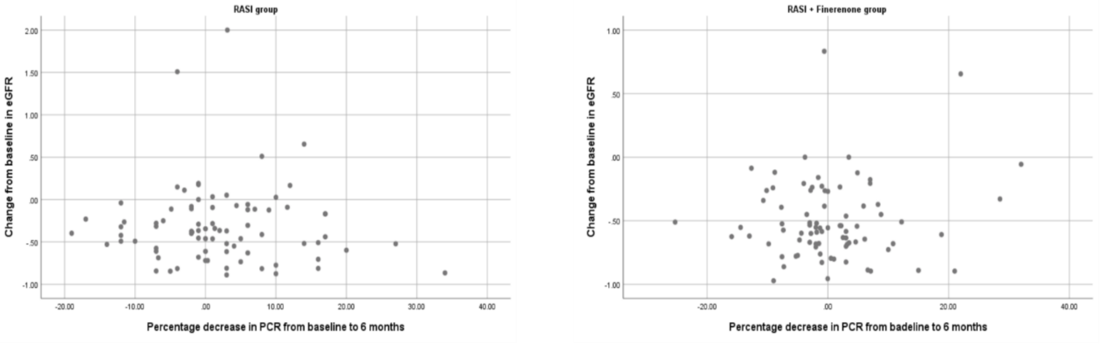

Supplement: sfaf125_Supplemental_Files [file sfaf125_supplemental_files.zip › Supplementary figure.pdf]
