# Supplementary material for: Efficacy and safety of finerenone in IgA nephropathy: an observational multicentre study
Source: Clin Kidney J. 2025 Apr 28;18(5):sfaf125. doi: 10.1093/ckj/sfaf125 (PMC12067071; doi:10.1093/ckj/sfaf125)
Supplement: sfaf125_Supplemental_Files [file sfaf125_supplemental_files.zip › Supplementary table.docx]

**Table S1:** Baseline characteristics of IgAN patients at the enrollment

|  | **RASI group(n=144)** | **RASI + Finerenone group(n=97)** | ***P*** |
| --- | --- | --- | --- |
| Age, y | 39.00(33.00,47.00) | 33.00(29.00,45.00) | 0.191 |
| Sex, male | 60(41.7%) | 61(62.9%) | 0.001 |
| Body mass index, kg/m^2^ | 21.25(18.14,22.10) | 27.72(24.30,29.75) | 0.024 |
| Hypertension, yes | 60(41.7%) | 43(44.3%) | 0.621 |
| Hypertension hierarchy, 90/60mmHg<BP<130/80(n) | 138(95.8%) | 91(93.8%) | 0.677 |
| Diabetes Mellitus. Yes | 15(10.4%) | 9(9.3%) | 0.832 |
| Serum creatinine, μmol/L | 88.00(71.07,125.00) | 108.00(79.00,131.00) | 0.012 |
| eGFR, mL/min/1.73 m^2^ | 79.00(60.00,100.00) | 64.80(51.84,85.00) | 0.006 |
| PCR, g/g | 0.78(0.43,1.09) | 0.73(0.51,1.2) | 0.377 |
| Albumin, g/L | 41.35±3.52 | 42.14±3.17 | 0.083 |
| Potassium, mmol/L | 4.12(3.91,4.36) | 4.11(3.93,4.41) | 0.655 |
| Blood glucose, mmol/L | 4.98±0.94 | 5.10±0.82 | 0.370 |
| Sodium, mmol/L | 140.31(138.88,141.80) | 141.00(139.10,142.00) | 0.086 |
| Hemoglobin, g/L | 137.04±18.65 | 138.90±18.14 | 0.455 |
| Hematocrit, % | 41.10±5.22 | 42.07±5.13 | 0.163 |
| Uric acid, μmol/L | 351.59±92.61 | 355.33±80.62 | 0.752 |
| Oxford classification |  |  |  |
| Mesangial hypercellularity, M1 | 70(48.6%) | 45(46.4%) | 0.754 |
| Endocapillary hypercellularity, E1 | 15(10.4%) | 9(9.3%) | 0.921 |
| Segmental glomerulosclerosis, S1 | 106(73.6%) | 73(75.3%) | 0.601 |
| Tubular atrophy/interstitial fibrosis |  |  | 0.903 |
| T1 | 36(25.0%) | 26(26.8%) |  |
| T2 | 3(2.1%) | 2(2.06%) |  |
| Cellular/ fibrocellular crescents |  |  | 0.249 |
| C1 | 44(30.6%) | 33(34.0%) |  |
| C2 | 5(3.5%) | 2(2.1%) |  |
| Therapy with SGLT2 inhibitors | 61(42.4%) | 49(50.5%) | 0.053 |
| Previous corticosteroid/immunosuppressive therapy | 60(41.7%) | 44(45.4%) | 0.162 |

Abbreviations: eGFR, estimated glomerular filtration rate; PCR, protein-to-creatinine ratio; HBP, high blood pressure; SGLT2 inhibitors, Sodium-Glucose Cotransporter 2 inhibitor.

Age, body mass index, serum creatinine, PCR are presented as median with interquartile range. Albumin, eGFR, potassium, sodium, blood glucose, hemoglobin, hematocrit, uric acid are presented as mean ± standard deviation. Categorical data as count and percentage.

**Table S2:** Baseline characteristics of IgAN patients who received systemic immunosuppressive therapy before in the full analysis set.

|  | **RASI group(n=36)** | **RASI +Finerenone(n=40)** | ***P*** |
| --- | --- | --- | --- |
| Time to discontinuation immunosuppressant, months | 12.77(20.19) | 16.19(15.92) | 0.310 |
| Age, y | 34.50(28.00,39.00) | 35.00(30.00,41.00) | 0.704 |
| Sex, male | 15(41.7%) | 18(45.0%) | 0.770 |
| Body mass index, kg/m^2^ | 24.24±3.35 | 24.79±2.74 | 0.435 |
| Hypertension, yes | 15 (41.7%) | 19 (47.5%) | 0.610 |
| Hypertension hierarchy, 90/60mmHg<BP<130/80(n) | 34 (94.4%) | 38 (95.0%) | 0.914 |
| Diabetes Mellitus. Yes | 13 (36.1%) | 17 (46.0%) | 0.393 |
| Serum creatinine, μmol/L | 100.95±33.60 | 99.91±32.69 | 0.892 |
| eGFR, mL/min/1.73 m^2^ | 74.49±27.30 | 77.68±23.99 | 0.590 |
| PCR, g/g | 0.89 (0.57,1.18) | 0.74 (0.62,1.29) | 0.743 |
| Albumin, g/L | 41.40±3.23 | 42.33±3.52 | 0.234 |
| Potassium, mmol/L | 4.19±0.37 | 4.14±0.38 | 0.605 |
| Blood glucose, mmol/L | 4.77±0.77 | 4.29±0.63 | 0.068 |
| Sodium, mmol/L | 140.44±2.55 | 140.90±1.90 | 0.373 |
| Hemoglobin, g/L | 139.73±19.03 | 139.30±17.90 | 0.919 |
| Hematocrit, % | 41.72±5.22 | 42.16±4.92 | 0.702 |
| Uric acid, μmol/L | 359.85±84.32 | 352.43±84.43 | 0.703 |
| Biopsy time, years | 2.90 (2.00,5.10) | 3.00 (2.00,4.00) | 0.811 |
| Oxford classification |  |  |  |
| M, M1 | 20 (55.6%) | 17 (42.5%) | 0.256 |
| E, E1 | 5 (13.9%) | 5 (12.5%) | 0.858 |
| S, S1 | 27 (75.0%) | 31 (77.5%) | 0.798 |
| T |  |  | 0.408 |
| T1 | 10 (27.8%) | 15 (37.5%) |  |
| T2 | 1 (2.8%) | 0 (0.0%) |  |
| C |  |  | 0.609 |
| C1 | 10 (27.8%) | 12 (30.0%) |  |
| C2 | 0 (0.0%) | 1 (2.5%) |  |
| Therapy with SGLT2 inhibitors | 13 (36.1%) | 17 (46.0%) | 0.393 |
| Duration of medication, months | 10.00 (5.5,18.30) | 9.00 (4.50,17.40) | 0.631 |
| Therapy of ACEI/ARB |  |  |  |
| >=50% of maximum labelled dose | 19 (52.78%) | 20 (50.00%) | 0.809 |
| Duration of medication, months | 12.00 (4.50,21.75) | 10.50 (3.00,21.50) | 0.821 |

Abbreviations: eGFR, estimated glomerular filtration rate; PCR, protein-to-creatinine ratio; HBP, high blood pressure; SGLT2 inhibitors, Sodium-Glucose Cotransporter 2 inhibitor; M, Mesangial hypercellularity; E, Endocapillary hypercellularity; S, Segmental glomerulosclerosis; T, Segmental glomerulosclerosis; C, Cellular/fibrocellular crescents; ACEI, angiotensin-converting enzyme inhibitor; ARB, angiotensin II receptor blocker.

Age, body mass index, biopsy time, serum creatinine, eGFR, PCR, Potassium, Sodium are presented as median with interquartile range. Albumin, blood glucose, hemoglobin, hematocrit, uric acid are presented as mean ± standard deviation. Categorical data as count and percentage.

**Table S3:** Baseline characteristics of IgA patients with or without combined SGLT2 inhibitors in the full analysis set.

|  | **RASI group (n=49)** | **RASI +S group (n=40)** | **RASI +F group (n=48)** | **RASI +S +F group (n=41)** | ***P*** |
| --- | --- | --- | --- | --- | --- |
| Age, y | 37.00(30.00,45.00) | 39.00(33.00,48.00) | 35.00(30.00,48.00) | 38.00(34.00,46.00) | 0.487 |
| Sex, male | 24(49.0%) | 16(40.0%) | 25(52.1%) | 25(61.0%) | 0.301 |
| BMI, kg/m2 | 24.49(21.50,26.14) | 24.32(22.94,26.63) | 23.29(21.50,25.76) | 24.64(22.33,26.15) | 0.251 |
| Hypertension, yes | 23(46.9%) | 17（42.5%） | 18(37.5%) | 21(51.2%) | 0.597 |
| Hypertension hierarchy, 90/60mmHg<BP<130/80(n) | 46(93.9%) | 38(95.0%) | 47(97.9%) | 36(87.8%) | 0.549 |
| Diabetes Mellitus, yes | 2(4.1%) | 5(12.5%) | 4(8.3%) | 4(9.8%) | 0.549 |
| Scr, μmol/L | 98.00(74.00,130.00) | 90.00(75.37,114.45) | 100.50(78.75,115.00) | 110.00(76.00,138.00) | 0.521 |
| eGFR, mL/min/1.73 m2 | 73.16±27.18 | 78.07±23.62 | 74.17±21.84 | 71.47±27.57 | 0.698 |
| PCR, g/g | 0.80(0.59,1.10) | 0.88(0.58,1.23) | 0.76(0.61,1.07) | 0.84(0.68,1.37) | 0.798 |
| Albumin, g/L | 41.40(38.70,43.70) | 42.85(40.12,44.23) | 41.82(38.98,43.92) | 42.40(40.60,44.32) | 0.160 |
| Potassium, mmol/L | 4.13±0.35 | 4.14±0.35 | 4.13±0.34 | 4.13±0.39 | 0.998 |
| Blood glucose, mmol/L | 4.71±0.42 | 5.19±0.87 | 4.69±0.56 | 4.99±0.69 | 0.098 |
| Sodium, mmol/L | 140.50±2.39 | 140.88±1.90 | 140.08±1.98 | 141.73±2.61 | 0.012 |
| Hemoglobin, g/L | 136.00(124.00,149.00) | 139.03(123.00,151.00) | 142.00(128.00,155.00) | 138.50(128.00,147.00) | 0.785 |
| Hematocrit, % | 41.30±5.15 | 41.93±4.96 | 41.92±5.66 | 41.80±5.36 | 0.931 |
| Uric acid, μmol/L | 360.24±86.00 | 350.23±89.52 | 373.90±97.32 | 334.04±86.26 | 0.261 |
| Biopsy time, years | 3.40(2.00,5.30) | 3.65(2.60,9.00) | 3.00(1.50,4.10) | 3.50(2.00,6.00) | 0.105 |
| Oxford classification |  |  |  |  |  |
| M, M1 | 25(51.0%) | 20(50.0%) | 22(45.8%) | 18(43.95%) | 0.895 |
| E, E1 | 7(14.3%) | 4(10.0%) | 6(12.5%) | 8(19.5%) | 0.647 |
| S, S1 | 30(61.2%) | 29(72.5%) | 32(66.7%) | 31(75.6%) | 0.469 |
| T |  |  |  |  | 0.786 |
| T1 | 9(18.3%) | 11(27.5%) | 12(25.5%) | 12(29.3%) |  |
| T2 | 2(4.1%) | 0(0.0%) | 1(2.1%) | 1(2.4%) |  |
| C |  |  |  |  | 0.290 |
| C1 | 10(20.4%) | 13(32.5%) | 19(39.6%) | 12(29.3%) |  |
| C2 | 0(0.0%) | 0(0.0%) | 1(2.1%) | 0(0.0%) |  |
| Therapy with diuretics | 0 | 0 | 0 | 0 |  |
| Therapy of ACEI/ARB |  |  |  |  |  |
| >=50% of maximum labelled dose | 27(55.1%) | 26(66.7%) | 24(50.0%) | 24(58.5%) | 0.465 |
| Duration of medication, months | 15.00(5.00,24.00) | 5.00(3.00,17.00) | 6.00(3.00,14.50) | 14.00(4.00,26.00) | 0.017 |
| Previous immunosuppressive therapy | 23(46.9%) | 13(32.5%) | 21(43.7%) | 19(46.3%) | 0.512 |
| Time to discontinuation immunosuppressant, months | 6.00(3.50,10.00) | 12.00(4.00,12.00) | 10.00(5.00,13.00) | 14.00(10.00,24.00) | 0.124 |

Abbreviations: SGLT2, Sodium-Glucose Cotransporter 2 Inhibitor; RASI +S, RASI +SGLT2; RASI +F, RASI +finerenone; RASI +S +F group, RASI +SGLT2 +finerenone; BMI, body mass index; Scr, serum creatine; eGFR, estimated glomerular filtration rate; PCR, protein-to-creatinine ratio; M, Mesangial hypercellularity; E, Endocapillary hypercellularity; S, Segmental glomerulosclerosis; T, Segmental glomerulosclerosis; C, Cellular/fibrocellular crescents; ACEI, angiotensin-converting enzyme inhibitor; ARB, angiotensin II receptor blocker.

Age, BMI, Scr, PCR, biopsy time are presented as median with interquartile range. Albumin, eGFR, potassium, sodium, blood glucose, hemoglobin, hematocrit, uric acid are presented as mean ± standard deviation. Categorical data as count and percentage.

**Table S4:** Percentage of protein-to-creatinine ratio (PCR) reduction for different missing data imputation methods

|  | **RASI group (n=89)** | **RASI +finerenone group (n=89)** | ***P*** |
| --- | --- | --- | --- |
| Worst-case imputation | -36.1% (-40.5, -31.7) | -46.9% (-51.5, -42.2) | 0.001 |
| LOCF | -37.0% (-43.5, -30.5) | -43.7% (-50.2, -37.2 | <0.001 |
| Mean imputation | -35.1% (-39.5, -30.9) | -44.2% (-50.9, -38.4) | <0.001 |

Abbreviations: LOCF, Last Observation Carried Forward.

**Table S5**: Percentage change in PCR from baseline to month 6 in subgroup analyses in full analysis population.

| **Subgroup** | **RASI group(n=89)** | **RASI + Finerenone group(n=89)** | ***P*** |
| --- | --- | --- | --- |
| eGFR |  |  | 0.046 |
| eGFR>=60 | -32.4% (-40.7, -24.2) | -39.5% (-47.9, -30.9) | 0.042 |
| eGFR<60 | -32.4% (-45.6, -19.3) | -56.8% (-69.1, -44.6) | 0.034 |
| PCR |  |  | 0.013 |
| PCR>=1 | -37.7% (-50.5, -25.0) | -47.4% (-61.5, -33.3) | 0.603 |
| PCR<1 | -29.4% (-38.7, -20.0) | -44.2% (-53.1, -35.2) | 0.013 |

Abbreviations: eGFR, estimated glomerular filtration rate; PCR, protein-to-creatinine ratio. SGLT2 inhibitors: Sodium-Glucose Cotransporter 2 Inhibitor.

Percentage change in PCR is presented as median with interquartile range.

**Table S6**: The characteristics of IgA nephropathy at 6 month and changes from baseline after 6 months.

| **Parameter** | **RASI group(n=89)** | **RASI + Finerenone group(n=89)** |
| --- | --- | --- |
| Serum creatinine, umol/L |  |  |
| At 6 mo | 91.40(80.00,119.00) | 104.00(74.5,133.00) |
| Change from baseline | -8.60 | 5.00 |
| ***P*** value | 0.040 | 0.31 |
| eGFR, mL/min/ 1.73 m2 |  |  |
| At 6 mo | 76.98±29.82 | 73.21±31.64 |
| Change from baseline | 2.19±5.68 | -1.94±6.73 |
| ***P*** value | 0.044 | 0.123 |
| Albumin, g/L |  |  |
| At 6 mo | 42.30(38.10,44.10) | 41.10(39.20,43.50) |
| Change from baseline | 0.8 | -0.55 |
| ***P*** value | 0.060 | 0.268 |
| Potassium, mmol/L |  |  |
| At 6 mo | 4.18±0.40 | 4.31±0.37 |
| Change from baseline | 0.07±0.42 | 0.17±0.36 |
| ***P*** value | 0.064 | 0.03 |
| Sodium, mmol/L |  |  |
| At 6 mo | 140.10(129.30,155.30) | 140.14(126.22,154.60) |
| Change from baseline | 0.801 | 0.491 |
| ***P*** value | 0.569 | 0.017 |
| Hemoglobin, g/L |  |  |
| At 6 mo | 139.96±17.87 | 138.49±19.05 |
| Change from baseline | -0.04±1.34 | -2.51±13.40 |
| ***P*** value | 0.733 | 0.060 |
| Hematocrit, % |  |  |
| At 6 mo | 42.69±7.13 | 41.80±5.54 |
| Change from baseline | 1.03±6.56 | 0.01±1.81 |
| ***P*** value | 0.109 | 0.827 |
| Uric acid, umol/L |  |  |
| At 6 mo | 347.96±84.92 | 358.79±75.54 |
| Change from baseline | -15.38±7.86 | 9.30±7.36 |
| ***P*** value | 0.250 | 0.272 |

Abbreviations: eGFR, estimated glomerular filtration rate.

Serum creatinine is presented as median with interquartile range. Albumin, eGFR, potassium, sodium, hemoglobin, hematocrit, uric acid are presented as mean ± standard deviation

***P*** value indicates whether there is statistical significance between before and after treatment.
